# The Pharmacological Mechanism of Curcumin against Drug Resistance in Non-Small Cell Lung Cancer: Findings of Network Pharmacology and Bioinformatics Analysis

**DOI:** 10.1155/2022/5926609

**Published:** 2022-10-14

**Authors:** Han Xie, Guirui Huang, Jianhua Zou, Qianru Zhu, Zimao Liang, Xingxing Huang, Xiangyang Zhai, Ruonan Zhang, Bi Chen, Keshuai Li, Xinbing Sui, Lili Yu, Hongsheng Cui, Qibiao Wu

**Affiliations:** ^1^State Key Laboratory of Quality Research in Chinese Medicines, Faculty of Chinese Medicine, Macau University of Science and Technology, Wai Long, Macau; ^2^School of Pharmacy and Department of Medical Oncology, The Affiliated Hospital of Hangzhou Normal University, School of Medicine, Hangzhou Normal University, Hangzhou 311121, Zhejiang, China; ^3^Key Laboratory of Elemene Class Anti-Cancer Chinese Medicines, Hangzhou, China; ^4^Engineering Laboratory of Development and Application of Traditional Chinese Medicines, Hangzhou, China; ^5^Collaborative Innovation Center of Traditional Chinese Medicines of Zhejiang Province, Hangzhou Normal University, Hangzhou 311121, Zhejiang, China; ^6^Department of Respiratory, The Third Affiliated Hospital, Beijing University of Chinese Medicine, Beijing, China; ^7^Guangdong-HongKong-Macao Joint Laboratory for Contaminants Exposure and Health, Guangdong University of Technology, Guangzhou 510006, China; ^8^Zhuhai MUST Science and Technology Research Institute, Zhuhai 519000, Guangdong, China; ^9^Zhuhai Hospital of Integrated Traditional Chinese and Western Medicine, Zhuhai 519000, Guangdong, China

## Abstract

The pharmacological mechanism of curcumin against drug resistance in non-small cell lung cancer (NSCLC) remains unclear. This study aims to summarize the genes and pathways associated with curcumin action as an adjuvant therapy in NSCLC using network pharmacology, drug-likeness, pharmacokinetics, functional enrichment, protein-protein interaction (PPI) analysis, and molecular docking. Prognostic genes were identified from the curcumin-NSCLC intersection gene set for the following drug sensitivity analysis. Immunotherapy, chemotherapy, and targeted therapy sensitivity analyses were performed using external cohorts (GSE126044 and IMvigor210) and the CellMiner database. 94 curcumin-lung adenocarcinoma (LUAD) hub targets and 41 curcumin-lung squamous cell carcinoma (LUSC) hub targets were identified as prognostic genes. The anticancer effect of curcumin was observed in KEGG pathways involved with lung cancer, cancer therapy, and other cancers. Among the prognostic curcumin-NSCLC intersection genes, 20 LUAD and 8 LUSC genes were correlated with immunotherapy sensitivity in the GSE126044 NSCLC cohort; 30 LUAD and 13 LUSC genes were associated with immunotherapy sensitivity in the IMvigor210 cohort; and 12 LUAD and 13 LUSC genes were related to chemosensitivity in the CellMiner database. Moreover, 3 LUAD and 5 LUSC genes were involved in the response to targeted therapy in the CellMiner database. Curcumin regulates drug sensitivity in NSCLC by interacting with cell cycle, NF-kappa B, MAPK, Th17 cell differentiation signaling pathways, etc. Curcumin in combination with immunotherapy, chemotherapy, or targeted drugs has the potential to be effective for drug-resistant NSCLC. The findings of our study reveal the relevant key signaling pathways and targets of curcumin as an adjuvant therapy in the treatment of NSCLC, thus providing pharmacological evidence for further experimental research.

## 1. Introduction

Lung cancer is one of the most prevalent malignant tumors in the world. According to GLOBOCAN 2020, cancer incidence, and mortality estimates compiled by the International Agency for Research on Cancer, lung cancer is still the leading cause of cancer death, with an estimated 1.8 million people (18 percent). The mortality rate of lung cancer ranks first among all malignant tumors, while its incidence rate (11.4 percent) is lower than breast cancer [1]. According to the Registry of China's National Cancer Center, lung cancer ranks first in morbidity and mortality among numerous malignancies in China. It deserves the designation “cancer no. 1 killer” [2]. Non-small cell lung cancer (NSCLC) has the highest proportion of lung malignancies at 80%, and the absence of practical early-detection tools is one of the main reasons for its poor prognosis. Although comprehensive therapy with surgery, chemotherapy, radiation, targeted therapy, and immunotherapy has considerably improved the survival of lung cancer patients, however, these therapies exhibit drug resistance. Therefore, unraveling the mechanism of lung cancer and identifying new anticancer medications as adjuvant therapy with low toxicity and side effects is of considerable value.

Curcumin (1,7-bis (4-hydroxy-3-methoxyphenyl)-1,6-heptadiene-3,5-dione) is a potent component of turmeric (*Curcuma longa*), which was used in traditional Chinese medicine from ancient times [3]. Curcumin is commonly utilized for the treatment of various cancers (e.g., breast cancer and hematological cancers) [4]. It has been found to have anticancer activity in NSCLC [5, 6]. Increasing evidence showed that curcumin could enhance immune function via various mechanisms [7]. It was revealed that curcumin could enhance tumor antigen-specific T-cell induction in cancer cells [8]. Curcumin inhibited CSN5 expression in cancer cells and sensitized them to anti-CTLA4 therapy [9]. Additionally, curcumin could reverse resistance to targeted medicines. Curcumin overcomes primary gefitinib resistance in NSCLC cells by causing cell death related to autophagy [10]. Curcumin enhances gefitinib's sensitivity by inhibiting cell proliferation and suppressing clonogenic capacity in NCI-H1975 cells [11]. Curcumin can also enhance the effect of chemotherapy drugs. A previous study demonstrated that curcumin and doxorubicin work together more effectively in treating Hodgkin lymphoma [12].

Nowadays, the literature on combination treatments is far less developed than on individual therapies, and the associated targets are unknown. In this study, we systematically analyzed the multi-scale mechanism by which curcumin treats NSCLC as adjuvant therapy, combining drug prediction, network pharmacology, functional bioinformatics studies, and molecular docking. We do an enrichment analysis of the functional pathways involving the total number of curcumin and NSCLC intersecting genes and then look for linked prognostic genes. Among the prognostic genes, we mined the targets correlated with immunotherapy, chemotherapy, and targeted therapy sensitivity. This work will help identify promising essential signaling pathways and targets for curcumin's adjuvant therapy against NSCLC, providing critical pharmacological data for future experimental trials.

## 2. Material and Methods

### 2.1. Ethics Statement

The data used in this study were derived from open-source databases, so no ethics committee permission was required.

### 2.2. Curcumin Database Building

PubChem (https://pubchem.ncbi.nlm.nih.gov/) [13] was used to download the 2D structure, 3D structure, InChI, and canonical SMILES profiles of curcumin.

### 2.3. Analyses of Physicochemical Properties and Biological Activities

We uploaded the molecules to the Molinspiration property prediction tool (https://www.molinspiration.com), which analyzes molecular properties according to Lipinski's rule of five and reports on any violation of a particular property to determine the curcumin's drug-likeness and biological activity. Curcumin's standard SMILES profile was submitted to the section “Calculation of Molecular Properties and Bioactivity Score.” [14].

### 2.4. Collection of Curcumin-Related Targets

Curcumin-related pharmacological targets were obtained from the following databases: (1) Comparative Toxicogenomics Database (CTD, http://ctdbase.org/) [15], (2) Drug Gene Interaction Database (DGIdb, https://www.dgidb.org/) [16], (3) PharmMapper (http://www.lilab-ecust.cn/pharmmapper/) [17], (4) Chemical Association Networks (STITCH, http://stitch.embl.de/) [18], (5) Swiss Target Prediction (http://www.swisstargetprediction.ch/) [19], (6) Symptom Mapping (SymMap, symmap.org) [20], and (7) Traditional Chinese Medicine Systems Pharmacology Database and Analysis Platform (TCMSP, https://old.tcmsp-e.com/tcmsp.php) [21].

### 2.5. Collection of NSCLC-Related Targets

NSCLC-related targets were discovered by examining available transcriptome RNA-seq data from TCGA-lung adenocarcinoma (LUAD) and TCGA-lung squamous cell carcinoma (LUSC) [22] downloaded on February 24, 2022. Using the *R* package “limma” (version 4.1.0, https://www.r-project.org/), the profile of differentially expressed genes (DEGs) with criteria of adjusted *p* value 0.05 and |log_2_FC| > 1 was obtained. Volcano plots were used to depict DEGs, which were created using the *R* language packages “ggpubr” and “ggthemes.”

### 2.6. Targets of Curcumin against NSCLC Acquisition

Overlapped targets of curcumin and NSCLC (LUAD or LUSC) were identified using the Venn diagram tool (http://bioinformatics.psb.ugent.be/webtools/Venn/). Curcumin's target against NSCLC was the intersection of curcumin-related and NSCLC-related targets.

### 2.7. Enrichment Analyses for Intersection Targets

Through g:Profiler (https://biit.cs.ut.ee/gprofiler/gost), enrichment studies of Gene Ontology (GO) (containing molecular function, cellular component, and biological process) and Kyoto Encyclopedia of Genes and Genomes (KEGG) pathways of hub targets were conducted [23]. The organism was defined as “*Homo sapiens*,” and significantly enriched terms were those with a q value of 0.05 or more. Low-q value GO terms and pathway terms had a greater impact on curcumin against NSCLC, and the top 30 GO terms and pathway terms were ranked from low to high based on their q values.

### 2.8. The Prognosis Analysis of Curcumin-NSCLC Genes

R packages “limma,” “survival,” and “survminer” were used to analyze the prognosis. The route terms were displayed in the results, graded from low to high according to their adjusted *p* value. Genes with a *p* value less than 0.05 were considered prognostic curcumin-NSCLC intersection genes.

### 2.9. An Analysis of the Protein-Protein Interaction Network and Hub Targets

PPI networks give critical information for deciphering protein activities and system-level biological processes. As a result, we analyzed prognostic curcumin-NSCLC intersection genes and built the PPI network using the STRING 11.0b database (https://string-db.org/). The organism was defined as “*Homo sapiens*,” and a minimum interaction score of 0.4 was required [24]. Using the Cytoscape 3.8.0 software (https://cytoscape.org/), we visualized and analyzed the PPI network. By using the cytoHubba plug-in, the degree centrality (DC) median values were analyzed to determine the top 15 hub genes.

### 2.10. Immune Checkpoint, Chemotherapy, and Targeted Therapy Sensitivity Analysis Using Prognostic NSCLC-Curcumin Intersection Genes

Additionally, we explored changes in curcumin-NSCLC gene expression between immunotherapeutic responders and non-responders in two different immunotherapeutic cohorts (GSE126044 and IMvigor210) [25]. The GSE126044 cohort was associated with response to anti-PD-1 therapy in NSCLC and can be downloaded from https://www.ncbi.nlm.nih.gov/geo/query/acc.cgi. The IMvigor210 urothelial cancer data set was used to determine whether curcumin's anticancer effects are universal. The fully described software and data package can be downloaded from http://research-pub.gene.com/IMvigor210CoreBiologies/ using the Creative Commons 3.0 license. The data processing and visualization were carried out in *R* using the “limma,” “ggplot2,” and “ggpubr” packages. Using the CellMiner database (http://discover.nci.nih.gov/cellminer/), a correlation study was conducted between curcumin-NSCLC expression and commonly used chemotherapy and targeted therapy sensitivity [26]. The data processing and visualization were carried out in *R* using the “impute,” “limma,” “ggplot2,” and “ggpubr” packages.

### 2.11. Molecular Docking Verification of Curcumin and Targets

**Figure 1:**
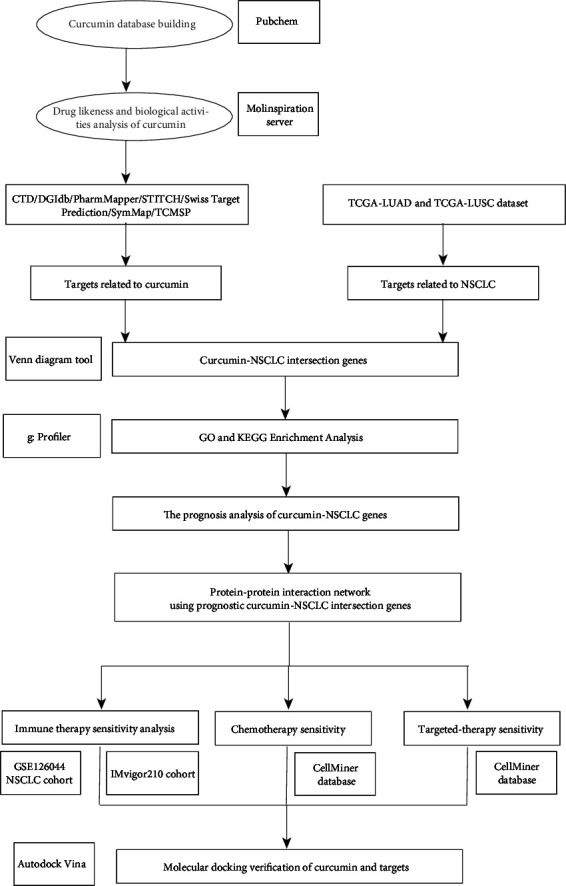
The flow diagram of this research shows strategies for identifying the pharmacological mechanism of curcumin against anticancer drug resistance based on network pharmacology and bioinformatics analysis.

The Protein Data Bank (PDB) database (https://www.rcsb.org/) [27] and AlphaFold Database (https://alphafold.ebi.ac.uk/) [28] were used to download the protein structures of targets. PubChem (https://pubchem.ncbi.nlm.nih.gov/) provided the curcumin structure and chemical formula. PyMOL (https://pymol.org/2/) removed water molecules and small molecule ligands from the large molecule protein. The small and large molecules were imported into AutoDock (Vina 1.5.6, http://autodock.scripps.edu/) for PDBQT file conversion, active pocket setting, and docking prediction [29] (Figure 1).

## 3. Results

### 3.1. Analysis of the Physicochemical Properties of Curcumin

We initially predicted the physicochemical properties of curcumin using Lipinski's rule of five to assess drug-likeness, which is crucial in the production and upgrading of pharmacological entities. The criteria of Lipinski's rule of five are as follows: logP ≤ 5, molecular weight (MW) ≤ 500 Da, number of hydrogen bond acceptors (n-ON) ≤ 10, and number of hydrogen bond donors (n-OHNH) ≤ 5 [30]. The criteria of topological polar surface area (TPSA) and percentage of absorption (%ABS) value are as follows: TPSA ≤ 140 Å [31] and ABS between 67% and 83% [32]. As shown in Table 1, the results showed that curcumin met the standard criteria, which means it has an optimal oral bioavailability.

### 3.2. Bioactivity Prediction of Curcumin

Curcumin's physiological role, as outlined in Table 2, may be mediated by several pathways, including interactions with GPCR ligands, ion channel modulators, kinase inhibitors, nuclear receptor ligands, protease inhibitors, and enzyme inhibitors. The data demonstrated that curcumin had a higher affinity for nuclear receptor ligands (nuclear receptor ligand > enzyme inhibitor > GPCR ligand > protease inhibitor > ion channel modulation > kinase inhibitor).

### 3.3. Target Identification of Curcumin and NSCLC Comorbidity

**Figure 2:**
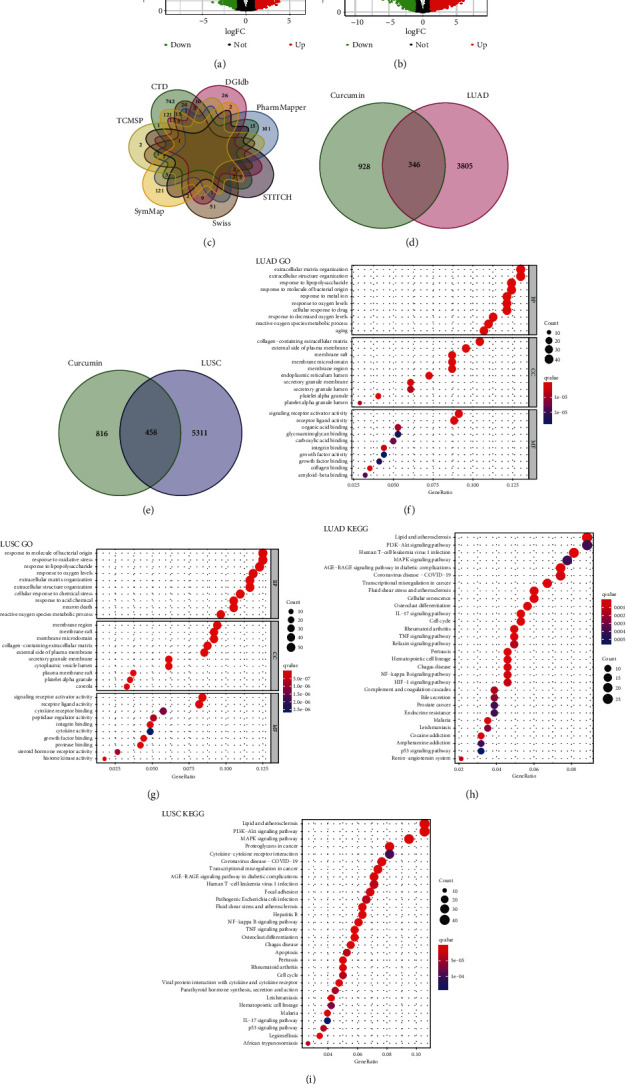
(a) Volcano plots of DEGs for LUAD patients. The abscissa represents log_2_FC and the ordinate indicates the −log_10_ (adjusted *p* value) of the genes. The red and green points, respectively, represent the upregulated and downregulated genes with the criteria of adjusted *p* value  < 0.05 and |log_2_FC| > 1. (b) Volcano plots of DEGs for LUSC patients. (c) The number of target genes related to curcumin from seven open-source databases. (d) Venn diagram depicting common target genes between LUAD and curcumin. (e) Venn diagram depicting common target genes between LUSC and curcumin. (f) Gene Ontology enrichment analysis results of curcumin-LUAD intersection genes according to q value. (g) Gene Ontology enrichment analysis results of curcumin-LUSC intersection genes according to q value. (h) KEGG pathway enrichment analysis identification results of curcumin-LUAD intersection genes according to q value. (i) KEGG pathway enrichment analysis identification results of curcumin-LUSC intersection genes according to q value.

We obtained 4151 LUAD and 5769 LUSC-related genes by differential expression analysis using the RNA-seq data from TCGA-LUAD (535 tumor patients and 59 healthy controls) and TCGA-LUSC (502 tumor patients and 49 healthy controls). Volcano plots are presented in Figures 2(a) and 2(b).

We then obtained curcumin-related targets from seven open-source databases: CTD, DGIdb, PharmMapper, STITCH, Swiss, SymMap, and TCMSP. We created a curcumin-related target collection by syndicating the expected results, and 1274 curcumin-related targets were obtained after duplications were removed and gene symbols were transferred (Figure 2(c)).

Finally, we acquired 346 curcumin-LUAD intersection genes and 458 curcumin-LUSC intersection genes, which were assessed using the Venn diagram (Figures 2(d) and 2(e)).

### 3.4. GO Enrichment Analysis

Curcumin-NSCLC intersection targets were submitted to g:Profiler. The top 30 terms of biological process (BP), cellular component (CC), and molecular function (MF) were ranked by q value, and the results of the GO analysis are shown in Figures 2(f) and 2(g). For LUAD, BP enrichment analysis commonly included extracellular matrix organization, extracellular structure organization, response to lipopolysaccharide, etc. CC enrichment analysis generally comprised collagen-containing extracellular matrix, external side of the plasma membrane, membrane raft, etc. MF enrichment analysis mainly involved signaling receptor activator activity, receptor-ligand activity, organic acid binding, etc. For LUSC, BP enrichment analysis primarily consisted of response to molecules of bacterial origin, response to oxidative stress, response to lipopolysaccharide, etc. CC enrichment analysis principally embraced membrane region, membrane raft, membrane microdomain, etc. MF enrichment analysis basically contained signaling receptor activator activity, receptor-ligand activity, cytokine receptor binding, etc. GO terms suggested that curcumin may regulate cell proliferation, immune responses, cell differentiation, cell proliferation, response to oxidative stress, and cytokine receptor binding to perform its therapeutic effects against NSCLC.

### 3.5. Pathway Enrichment Analysis

The results of the KEGG analysis of LUAD and LUSC are shown in Figures 2(h) and 2(i), and the lung cancer-related pathway, therapy-related pathways, and other cancer-type KEGG pathways are shown in Tables S1 and S2. The first lung cancer-related KEGG pathway is the IL-17 signaling pathway in LUAD, while the tumor necrosis factor (TNF) signaling pathway is in LUSC. The first therapy-related KEGG pathway in LUAD is the cell cycle, while the NF-kappa B signaling pathway is in LUSC. There are overlapping lung cancer-related (osteoclast differentiation) and therapy-related (NF-kappa B signaling pathway and MAPK signaling pathway) KEGG pathways in LUAD and LUSC in the top 3 KEGG pathways. Th17 cell differentiation and cytokine-cytokine receptor interaction are common immunotherapy-related KEGG pathways in LUAD and LUSC. Some KEGG pathways are related to other cancers such as leukemia, bladder cancer, and prostate cancer.

### 3.6. The Prognosis Analysis of Curcumin-NSCLC Genes

**Figure 3:**
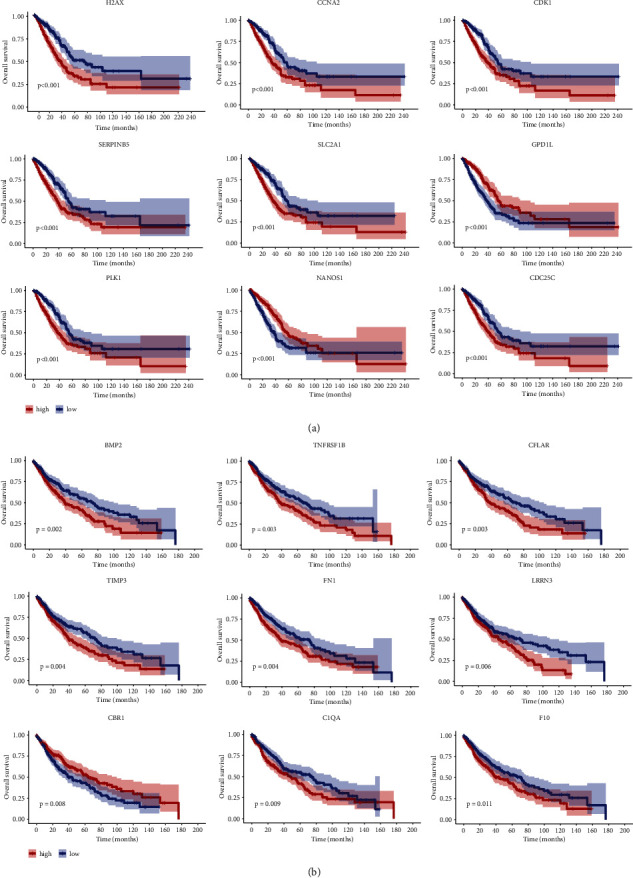
The prognosis analysis of curcumin-NSCLC intersection genes. (a) LUAD and (b) LUSC.

A total of 94 curcumin-LUAD intersection genes and 41 curcumin-LUSC intersection genes were identified with a *p* value < 0.05. With adjusted *p* value graded from low to high, Figure 3 lists the top 9 genes of LUAD and LUSC, respectively.

### 3.7. PPI Network Analysis

**Figure 4:**
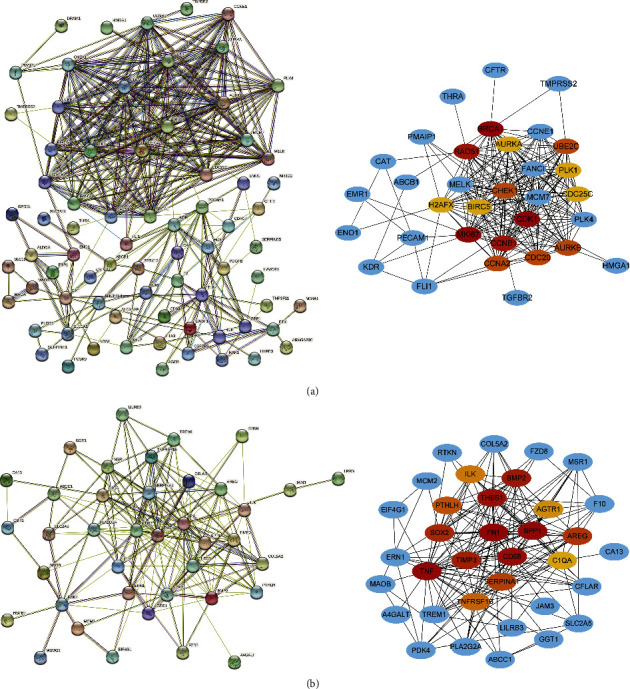
PPI network for prognostic curcumin-NSCLC intersection genes. Nodes and edges corresponded to hub targets and interactions among them. A node with a brighter color and a larger shape played a more significant part in combating LUAD or LUSC. The sizes and color shades of nodes are positively associated with degree values. (a) PPI network for hub targets of curcumin against LUAD. (b) PPI network for hub targets of curcumin against LUSC.

We examined 94 curcumin-LUAD intersection genes and 41 curcumin-LUSC intersection genes to assess curcumin's prognosis-related hub targets against LUAD or LUSC. The results are ranked from low to high according to their DC values. The hub targets were those with the top 15 DC values, and 15 LUAD hub targets and 15 LUSC hub targets were found. In Figure 4, a PPI network is visualized using the Cytoscape 3.8.0 program using the STRING 11.0b database.

### 3.8. Genomic and Clinical Data Sets with Immune Checkpoint, Chemotherapy, and Targeted Therapy Sensitivity

**Figure 5:**
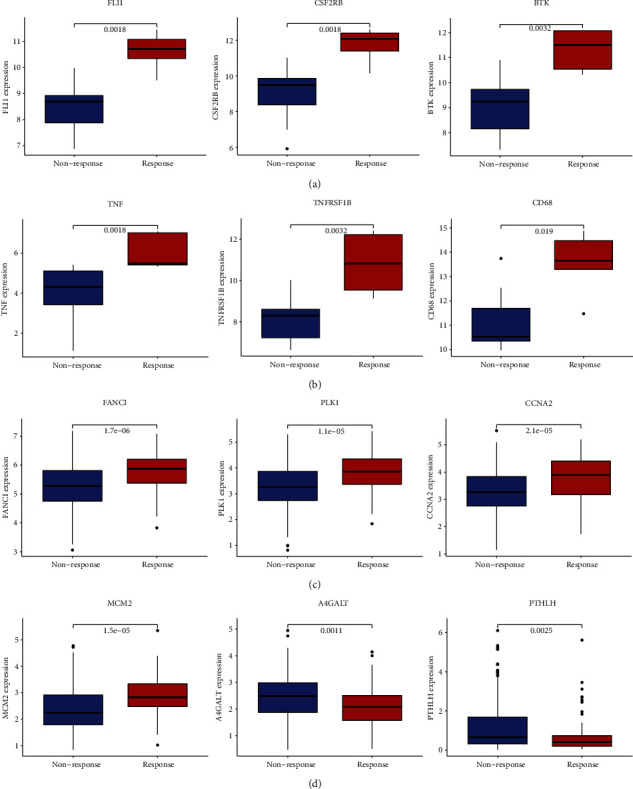
Immune checkpoint sensitivity exploration in the GSE126044 cohort and the IMvigor210 cohort using prognostic curcumin-NSCLC intersection genes. The x-coordinate represents response or non-response, and the y-coordinate shows the gene expression. (a) The top 3 related genes involved in LUAD in the GSE126044 cohort. (b) The top 3 related genes involved in LUSC in the GSE126044 cohort. (c) The top 3 related genes involved in LUAD in the IMvigor210 cohort. (d) The top 3 related genes involved in LUSC in the IMvigor210 cohort.

In the GSE126044 NSCLC cohort, immune therapy sensitivity was associated with 20 LUAD and 8 LUSC prognostic curcumin-NSCLC intersection genes (Figures 5(a), 5(b), and S1). Among them, LILRB3, LRRN3, and MAOB are genes that are both expressed in curcumin-LUAD and curcumin-LUSC intersection gene sets, and the same *p* values were verified (Figures S1A and S1B). FANCI, LRRN3, and CHEK1 are all negatively related to immune therapy response in LUAD (Figures S1B and S1D), but the remaining 17 genes are favorably linked to immune therapy response (Figures 5(a), S1A, and S1C). LRRN3 and RTKN are negatively connected to immune therapy response in LUSC (Figures S1B and S1F), whereas the remaining 6 genes have a positive relation with immune therapy response (Figures 5(b), S1A, and S1E). The results are ranked from low to high according to their adjusted *p* values which indicate the immunotherapy correlation. FLI1, CSF2RB, and BTK are the top 3 genes in LUAD (Figure 5(a)), and TNF, TNFRSF1B, and CD68 are the top 3 genes in LUSC (Figure 5(b)).

In the IMvigor210 cohort, immune therapy sensitivity was linked with 30 LUAD and 13 LUSC prognostic curcumin-NSCLC intersection genes (Figures 5(c), 5(d), and S2). MAOB and MIR22HG are genes that are both involved in curcumin-LUAD and curcumin-LUSC intersection gene sets, and the same *p* values were verified (Figure S2A). TGFBR2, MAOB, SLC18A2, MIR22HG, TNFSF11, TLR4, CFTR, GDF10, CDH17, and SERPINH1 are all negatively associated with immune treatment response in LUAD (Figures S2A and S2C), but the remaining 20 genes are favorably associated (Figures 5(c) and S2B). MCM2 and PUS1 were found to contribute positively to immune therapy response in LUSC (Figures 5(d) and S2D), whereas the remaining 11 genes may negatively affect immune therapy response (Figures 5(d), S2A, and S2E). FANCI, PLK1, and CCNA2 are the top 3 genes in LUAD (Figure 5(c)), and MCM2, A4GALT, and PTHLH are the top 3 genes in LUSC (Figure 5(d)).

**Figure 6:**
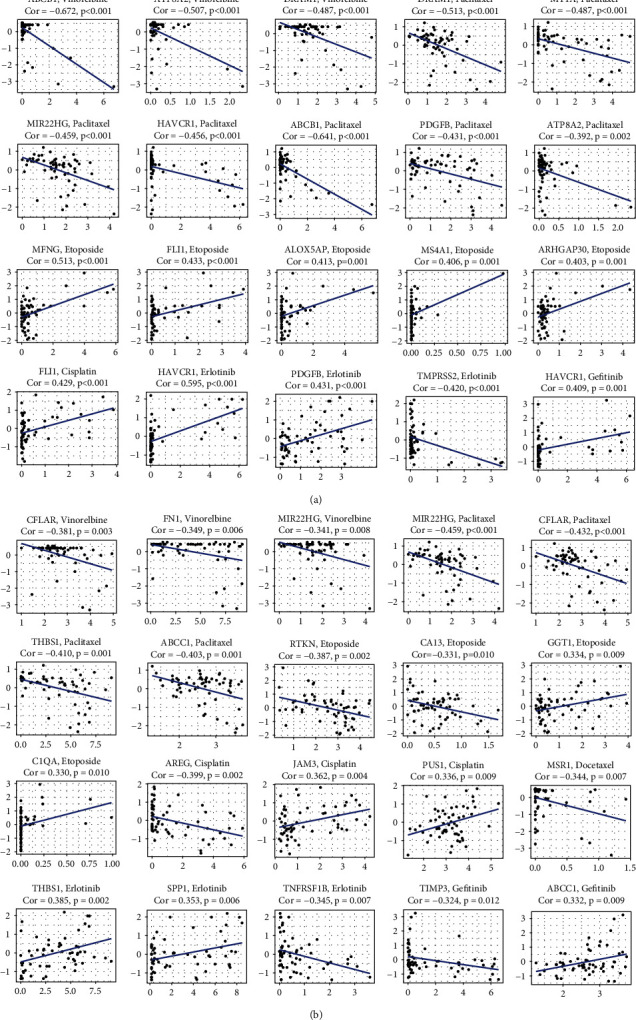
Transcription and drug pattern exploration in the NCI-60 cell line set from the CellMiner database using prognostic curcumin-NSCLC intersection genes. The x-coordinate is the amount of gene expression, and the y-coordinate is the z-scores. (a) LUAD. (b) LUSC.

In the CellMiner database, 12 LUAD and 13 LUSC genes were significantly related to chemosensitivity. Moreover, 3 LUAD and 5 LUSC genes were involved in the response to targeted therapy. In LUAD, ABCB1, ATP8A2, and DRAM1 are negatively related to vinorelbine. Paclitaxel has a negative correlation with DRAM1, MT1A, MIR22HG, HAVCR1, ABCB1, PDGFB, and ATP8A2. Etoposide is favorably linked to MFNG, FLI1, ALOX5AP, MS4A1, and ARHGAP30. FLI1 and cisplatin have a positive relationship. HAVCR1 and PDGFB have a favorable connection with erlotinib, while TMPRSS2 is negatively associated. A favorable link exists between gefitinib and HAVCR1 (Figure 6(a)).

In LUSC, CFLAR, FN1, and MIR22HG are unfavorably linked to vinorelbine response. MIR22HG, CFLAR, THBS1, and ABCC1 are linked to a poor response to paclitaxel. RTKN and CA13 adversely affect etoposide response, while GGT1 and C1QA have a favorable correlation. AREG has an inhibitory effect on cisplatin, while JAM3 and PUS1 play a favorable role. MSRI is negatively related to response to docetaxel. Erlotinib shows a positive relationship with THBS1 and SPP1 and an inverse connection with TNFRSF1B. TIMP3 is negatively correlated with gefitinib response, while ABCC1 is positively associated with gefitinib (Figure 6(b)).

### 3.9. Molecular Docking Analysis

**Figure 7:**
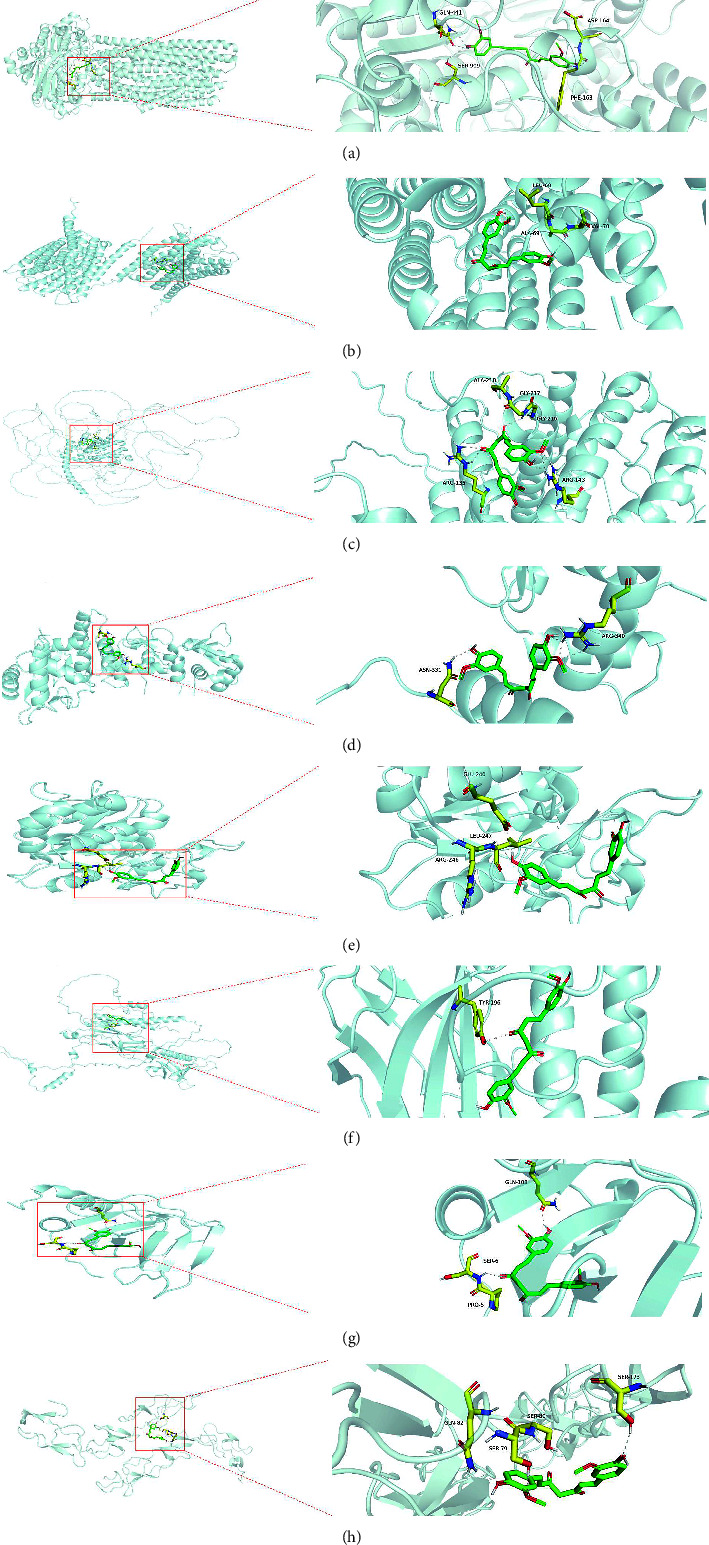
Detailed target compound interactions in the docking simulation. The green part represents curcumin, and the yellow part represents the amino acid residues of the target. (a) ABCB1 protein. (b) ALOX5AP protein. (c) ARHGAP30 protein. (d) FLI1 protein. (e) MFNG protein. (f) RTKN protein. (g) TIMP3 protein. (h) TNFRSF1B protein.

We selected key genes linked to prognosis, immunotherapy sensitivity prediction, and chemotherapy (or targeted therapy) sensitivity prediction and some were related to PPI findings as shown in Table 3. The molecular docking between the key targets (ABCB1, ALOX5AP, ARHGAP30, FLI1, MFNG, RTKN, TIMP3, and TNFRSF1B) and their corresponding active molecules was undertaken to validate the study results above. The chemicals were observed entering the active protein pocket using AutoDock software. Their affinity (Kcal/mol) was utilized to determine the degree of ligand binding to the receptor protein (the results are shown in Figure 7). The small molecule ligand can spontaneously bind to the protein receptor when the binding energy is 0 kJmol. If the binding energy is less than -5.0 kJmol, one of the two has a better binding ability. Table 3 also demonstrates the binding free energy.

## 4. Discussion

Lung cancer is one of the most often encountered malignant tumors worldwide. Chemotherapy, immunotherapy, and targeted therapy are available to restrict the malignant growth of cancer cells, despite their drug resistance. The process of drug resistance in tumors is complicated; involves physiological, pathological, endogenous, and external variables; and is frequently shared by many components. Traditional Chinese medicine therapy could benefit since it consists of numerous components, several targets, and various connections. Curcumin has a long history of anticancer usage, although its combination with conventional cancer treatments and its target are relatively unknown. As such, this study aims to determine the underlying mechanism of action of curcumin as adjuvant therapy in the treatment of NSCLC using network pharmacology analysis.

To better understand how curcumin exerts its therapeutic effects against LUAD or LUSC, GO analysis indicated that curcumin can affect cell proliferation, immunological responses, cell differentiation, cell proliferation, response to oxidative stress, and cytokine receptor binding. Curcumin's anticancer effect was observed in KEGG pathways involved with lung cancer (including IL-17 signaling pathway, TNF signaling pathway, osteoclast differentiation, etc.), cancer therapy (cell cycle, NF-kappa B signaling pathway, MAPK signaling pathway, Th17 cell differentiation, cytokine-cytokine receptor interaction, etc.), and other cancers (leukemia, bladder cancer, prostate cancer, etc.).

The cytokine family of IL-17 and its receptors are vital for normal host immune responses. Their dysregulated expression has been linked to a variety of human diseases, most notably cancer and inflammation. IL-17B and IL-17RB are the major protagonists in the development of tumors [33].

One of the crucial strategies for triggering apoptosis is death receptors, which are grouped in the TNF superfamily and comprise ligands like TNF-related apoptosis-inducing ligand (TRAIL) and Fas ligand (FasL). These ligands initiate apoptosis by interacting with their particular membrane receptors. The TNF superfamily is known to have 19 ligands and 29 receptors that function in various bodily processes such as inflammation, apoptosis, proliferation, and invasion [34].

Aberrant activity of bone-resorbing osteoclasts plays a crucial role in developing osteoporosis and cancer bone metastasis. An important step in osteolytic bone metastasis is osteoclastogenesis. The most typical site of metastasis in patients with lung cancer is the bone. In the later stages, up to 60–75% of these patients experienced bone metastases [35].

In recent years, knowledge of the cell cycle regulators like Rb and p53 illustrates their roles as onco-suppressive factors. It suggests a link between the regulation of the cell cycle and the initiation of carcinogenesis. Rapid and uncontrolled cell division, higher survival due to dedifferentiation processes, and an accumulation of genetic changes are all characteristics of neoplasms [36].

Activating the NF-kappa B signaling pathway is part of innate immunity when the body is under physical or oxidative stress, and it is linked to the process of inflammation. NF-kappa B activation is a component in the immune system's identifying and eliminating abnormal cells. This seems to be especially true during acute inflammatory events when complete NF-kappa B activation is linked to a significant degree of cytotoxic immune cell activity against cancer cells. Tumor immunosurveillance is another term that relates to the immune system's antitumorigenic activity, in which NF-kappa B plays a significant role [37].

The MAPK pathway regulates numerous biological processes, such as cell cycle activation, apoptosis, survival, and differentiation. Some studies have suggested that the AMPK and MAPK3/1 pathways may be biological indicators and possible targets for cancer therapy based on metabolic changes [38].

Numerous studies have shown that the Th17/Treg ratio and cytokine imbalance may play a role in the development of lung cancer. Restoring a normal cytokine network and Th17/Treg balance may help to improve the clinical response because Tregs and Th17 cells are implicated in the maintenance of the inflammatory immune response in lung cancer [39].

Cytokine-cytokine receptor interaction pathway is closely associated with the immune microenvironment. Cytokines are released glycoproteins that act as intermediaries between cells, boosting cellular division, proliferation, and apoptosis. On the other hand, cytokines released by tumors might encourage the recruitment of immune-suppressive cells, leading to the spread of the original tumor [40].

Some KEGG pathways are related to other cancers such as leukemia, bladder cancer, and prostate cancer. Moreover, in the IMvigor210 cohort, immune therapy sensitivity was linked with 30 LUAD and 13 LUSC genes. Hence, curcumin can also play a role in several cancers and even regulate the efficacy of immunotherapy.

Curcumin was determined to have multi-target overall control qualities after establishing a drug-prognostic drug resistance target to improve tumor drug resistance and analyze the biological process and signaling pathway involved in the action of curcumin. When studying the role of curcumin acting on immunotherapy resistance, we found that it works primarily through increasing immune cell production, modulating the inflammatory response, and activating receptors. FLI1, CSF2RB, and BTK are the top 3 genes in LUAD. FLI1 is an ETS family transcription factor that functions in hematopoiesis and other developmental pathways in both nonimmune and immune cell types. FLI1 deletion significantly increased antitumor immunity [41]. The CSF2RB is a shared beta-chain receptor required to activate the IL-3, IL-5, and GM-CSF receptors. CSF2RB was discovered to be connected with clinical characteristics of triple-negative breast cancer using the WGCNA as an immune-related hub gene [42]. BTK, a member of the TEC family of kinases, has recently been identified as a promising target for cancer therapy because of its critical role in B-cell activity and malignancy [43]. TNF, TNFRSF1B, and CD68 are the top 3 genes in LUSC. TNF was a significant mediator of inflammation associated with malignancy and has been discovered as a crucial player in the cytokine network outside of the cancer area. It promotes primary tumor growth, assists metastatic spread, and regulates the amount and type of leukocyte infiltration and angiogenesis in cancer [44]. The tumor necrosis factor receptor superfamily (TNFRSF), often known as TNFR2, includes TNFRSF1B. As one of the main TNF receptors, TNFRSF1B is involved in the control of tissue regeneration [45]. CD68, alone or in combination with other tumor-associated macrophage markers, had a high predictive value for survival in cancer patients [46].

Combined with chemotherapy, curcumin may work through autophagy, apoptosis, angiogenesis, drug uptake, the tumor microenvironment, microsatellite instability, pH homeostasis, interaction with miRNAs, immune cell differentiation, and cytoskeleton organization. In LUAD, ABCB1, ATP8A2, and DRAM1 are associated with vinorelbine and paclitaxel resistance. Previously published research established that the PCAT1/miR-129/ABCB1 axis confers chemoresistance on NSCLC [47]. The ATP8A2 protein is a member of the P4 ATPase family of proteins and a catalytic component of the P4-ATPase flippase complex. It catalyzes the hydrolysis of ATP involved in the transport of aminophospholipids from the outer to the inner leaflets of diverse membranes and ensures that the phospholipids maintain an asymmetric distribution [48]. DRAM1 induces autophagy and is downregulated in a variety of human cancers [49]. MT1A, MIR22HG, HAVCR1, and PDGFB were linked with paclitaxel resistance. MT1A is linked to the metabolism of trace elements [50]. MIR22HG is a compound linked to paclitaxel. As a host gene involved in the development and spread of cancer, MIR22HG has been demonstrated to exhibit competitive endogenous RNA (ceRNA) behavior, take part in signaling networks, and interact with proteins and miRNAs [51]. HAVCR1 is overexpressed in various malignancies and interacts with crucial tight junction molecules [52]. PDGFB is required to recruit pericytes with PDGF receptors to blood arteries. Because platelet granules are a crucial source of PDGFB and are constantly activated in the tumor microenvironment, they can expose tumors to a range of growth factors [53]. MFNG, ALOX5AP, MS4A1, and ARHGAP30 are related to etoposide resistance. MFNG encodes an O-fucosylpeptide 3-N-acetylglucosaminyltransferase that is known to alter EGF repeats in the Notch extracellular domain. It is overexpressed in claudin-low breast cancer and acts as an oncogene [54]. ALOX5AP is a gene involved in ferroptosis [55]. MS4A1, sometimes referred to as CD20, is a member of the MS4A family of genes. MS4A1 (CD20) is a critical marker of B-cell development and a therapeutic target in lymphoma [56]. ARHGAP30 is a compound that is linked to etoposide. ARHGAP30 is involved in controlling cytoskeleton organization and cell adhesion. ARHGAP30 suppresses lung cancer by inhibiting Wnt/-catenin signaling [57]. FLI1 is associated with etoposide and cisplatin resistance, and its function has been described previously.

In LUSC, CFLAR is associated with resistance to vinorelbine and paclitaxel. CFLAR(L), also known as c-FLIPL, is a key antiapoptotic protein in mammalian cells that suppresses caspase 8 activation [58]. FN1 is related to the resistance to vinorelbine. FN1 is a glycoprotein found in several cell types and involved in many processes like cell adhesion and migration. FN1 has been implicated in the regulation of lung cancer [59]. MIR22HG is associated with vinorelbine and paclitaxel resistance. The function of MIR22HG has been discussed previously. THBS1 is associated with resistance to erlotinib and paclitaxel. THBS1 is a secreted protein that acts in the tumor microenvironment to regulate antitumor immunity, stimulate tumor cell migration, and regulate the activities of extracellular proteases and growth factors. THBS1 also exerts a strong inhibitory effect on angiogenesis [60]. ABCC1 is related to resistance to paclitaxel. The ABC transporters are transmembrane proteins involved in the transit of physiologically significant substrates like amino acids and cholesterol. The overexpression of ABC transporters has been shown to decrease drug uptake and boost drug efflux, thus leading to a low drug density in the cytoplasm and resulting in decreased medication efficacy and eventually drug resistance [61]. RTKN is associated with resistance to etoposide. The RTKN gene was found to encode an effector for the Rho protein, which is essential for controlling cell proliferation [62]. CA13 has been identified as a member of the CA family that is required for pH homeostasis [63]. GGT1 is associated with resistance to etoposide. GGT1 is an enzyme that appears at the cell surface and modulates glutathione catabolism [64]. C1QA is part of C1q receptors and is related to resistance to etoposide. It has been demonstrated that C1q, the first part of the C system, increases the adhesion, motility, proliferation, angiogenesis, and metastasis of cancer cells, hence promoting the formation of tumors. C1q is a protein that serves as an extracellular matrix in the tumor microenvironment and promotes the growth and spread of tumors [65]. AREG is associated with resistance to cisplatin. AREG is one of the epidermal growth factor receptor (EGFR) ligands. AREG is also involved in cancer treatment resistance [66]. JAM3 is associated with resistance to cisplatin. JAM3 is a transcription factor that regulates adhesion and transmigration [67]. PUS1 is related to the resistance to cisplatin. PUS1 is a nuclear and mitochondrial enzyme that converts uridine to pseudouridine at various cytosolic and mitochondrial transfer RNA positions, thereby increasing the efficiency of protein synthesis in both compartments [68]. MSR1 is associated with resistance to docetaxel. MSR1 repeats, which are related to microsatellites and function as global regulators of gene expression, have been related to the formation of a number of serious clinical disorders [69].

When combined with EGFR-tyrosine kinase inhibitors (EGFR-TKIs), curcumin operates through tight junctions, growth factors, tissue regeneration, and medication uptake. HAVCR1, PDGFB, and TMPRSS2 are three significant genes associated with EGFR-TKI resistance in LUAD. HAVCR1 and PDGFB were discussed previously. TMPRSS2 is an androgen-regulated serine protease expressed mainly on the surface of the prostate epithelium [70]. THBS1, SPP1, TNFRSF1B, ABCC1, and TIMP2 are the significant genes associated with EGFR-TKI resistance in LUSC. THBS1 is previously described. SPP1, also known as osteopontin-like protein, is a released glycophosphoprotein. SPP1 contributes to the development of the second-generation EGFR-TKIs resistance in NSCLC [71]. TNFRSF1B and ABCC1 were discussed previously. TIMP2 is an MMP inhibitor that is expressed ubiquitously in normal tissues. MMPs are a class of zinc-dependent endopeptidases that play a critical role in the degradation and turnover of the ECM [72].

It is not difficult to conclude from the preceding data that curcumin has the potential to be developed as an additional medication for the treatment of NSCLC. Additionally, we mined targets that had not been empirically validated. Therefore, our research is of some significance.

## 5. Conclusion

Curcumin regulates drug sensitivity in NSCLC by interacting with cell cycle, NF-kappa B, MAPK, Th17 cell differentiation signaling pathways, etc. Curcumin in combination with immunotherapy, chemotherapy, or targeted drugs has the potential to be effective for drug-resistant NSCLC. The findings of our study reveal the relevant key signaling pathways and targets of curcumin as an adjuvant therapy in the treatment of NSCLC, aiming to provide pharmacological evidence for further experimental studies.

## Figures and Tables

**Table 1:** Physicochemical properties of curcumin evaluated by Molinspiration.

Compound	%ABS	miLogP	TPSA (Å)	n-atoms	MW	n-ON	n-OHNH	n-violations	n-ROTB	MV
Standard criteria	67–83	<5	≤140		<500	<10	<5	≤1	≤10	
Curcumin	76.89	2.30	93.07	27	368.38	6	2	0	8	332.18

%ABS, percentage of absorption; miLogP, the logarithm of partition coefficient between n-octanol and water; TPSA, topological polar surface area; n-atoms, number of atoms; MW, molecular weight; n-ON, number of hydrogen bond acceptors; n-OHNH, number of hydrogen bond donors; n-violations, number of Lipinski's rule-of-five violation; n-ROTB, number of rotatable bonds; MV, molecular volume.

**Table 2:** Bioactivity scores of curcumin based on Molinspiration cheminformatics.

Compound	GPCR ligand	Ion channel modulator	Kinase inhibitor	Nuclear receptor ligand	Protease inhibitor	Enzyme inhibitor
Curcumin	−0.06	−0.20	−0.26	0.12	−0.14	0.08

A bioactivity score of >0 represents promising activity, a bioactivity score between −5.00 and 0.00 represents moderate activity, and a bioactivity score of ≤ -5.0 represents no activity.

**Table 3:** NSCLC drug sensitivity-related genes and their key components, target molecular docking information.

Diseases	Gene	Prognosis	Prediction of immunotherapy sensitivity (GSE126044 NSCLC cohort)	Prediction of chemotherapy or targeted therapy sensitivity (CellMiner)	Protein-protein interaction	ID	Binding free energy (kcal/mol)
LUAD	ABCB1	Yes	Yes	Yes	No	CID: 969516	−7.7
LUAD	MFNG	Yes	Yes	Yes	No	CID: 969516	−6.6
LUAD	FLI1	Yes	Yes	Yes	No	CID: 969516	−6.8
LUAD	ALOX5AP	Yes	Yes	Yes	No	CID: 969516	−8.2
LUAD	ARHGAP30	Yes	Yes	Yes	No	CID: 969516	−6.0
LUSC	RTKN	Yes	Yes	Yes	No	CID: 969516	−6.7
LUSC	TIMP3	Yes	Yes	Yes	Yes	CID: 969516	−6.0
LUSC	TNFRSF1B	Yes	Yes	Yes	Yes	CID: 969516	−6.3

## Data Availability

The raw data utilized in this study are available publicly from the Comparative Toxicogenomics Database (CTD, http://ctdbase.org/), Drug Gene Interaction Database (DGIdb, https://www.dgidb.org/), PharmMapper (http://www.lilab-ecust.cn/pharmmapper/), Chemical Association Networks (STITCH, http://stitch.embl.de/), Swiss Target Prediction (http://www.swisstargetprediction.ch/), Symptom Mapping (SymMap, symmap.org), Traditional Chinese Medicine Systems Pharmacology Database and Analysis Platform (TCMSP, https://old.tcmsp-e.com/tcmsp.php), TCGA database (https://portal.gdc.cancer.gov/, cohort: TCGA-LUAD and TCGA-LUSC), GEO database (https://www.ncbi.nlm.nih.gov/geo/query/acc.cgi, GEO accession: GSE126044), IMvigor210CoreBiologies (http://research-pub.gene.com/IMvigor210CoreBiologies/), and CellMiner database (http://discover.nci.nih.gov/cellminer/).

## Abbreviations

%ABS: Percentage of absorption
BP: Biological process
CC: Cellular component
CTD: Comparative Toxicogenomics Database
DC: Degree centrality
DEGs: Differentially expressed genes
DGIdb: Drug Gene Interaction Database
ECM: Extracellular matrix
EGFR: Epidermal growth factor receptor
EGFR-TKIs: Epidermal growth factor receptor-tyrosine kinase inhibitors
GO: Gene Ontology
KEGG: Kyoto Encyclopedia of Genes and Genomes
LUAD: Lung adenocarcinoma
LUSC: Lung squamous cell carcinoma
MF: Molecular function
MW: Molecular weight
n-ON: Number of hydrogen bond acceptors
n-OHNH: Number of hydrogen bond donors
NSCLC: non-small cell lung cancer
PDB: Protein Data Bank
PPI: protein-protein interaction
TCMSP: Traditional Chinese Medicine Systems Pharmacology Database and Analysis Platform
TNF: Tumor necrosis factor
TNFRSF: Tumor necrosis factor receptor superfamily
TPSA: Topological polar surface area
n-atoms: Number of atoms.

## Supplementary Materials

Supplementary Materials Table S1. The lung cancer-related pathways, therapy-related pathways, and other cancer-related KEGG pathways in LUAD. Table S2. The lung cancer-related pathways, therapy-related pathways, and other cancer-related KEGG pathways in LUSC. Figure S1. Immune checkpoint sensitivity exploration in the GSE126044 NSCLC cohort using prognostic curcumin-LUAD and curcumin-LUSC intersection genes. Figure S2. Potential genes related to immunotherapy response in the IMvigor210 cohort using prognostic curcumin-LUAD and curcumin-LUSC intersection genes.
