# Supplementary figures and images for: The Pharmacological Mechanism of Curcumin against Drug Resistance in Non-Small Cell Lung Cancer: Findings of Network Pharmacology and Bioinformatics Analysis
Source: Evid Based Complement Alternat Med. 2022 Oct 14;2022:5926609. doi: 10.1155/2022/5926609 (PMC9586741; doi:10.1155/2022/5926609)

Figure S1

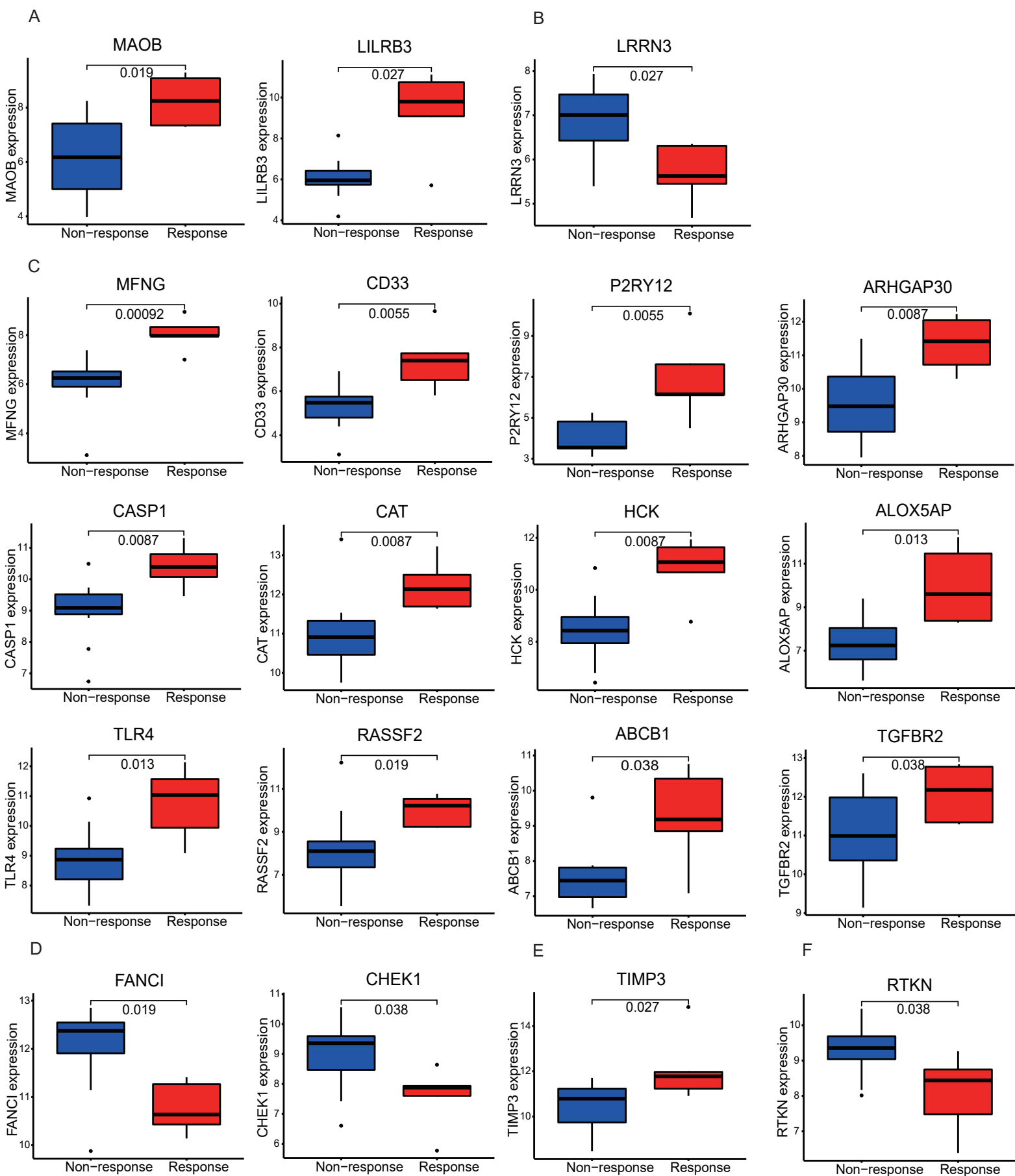

Figure S2

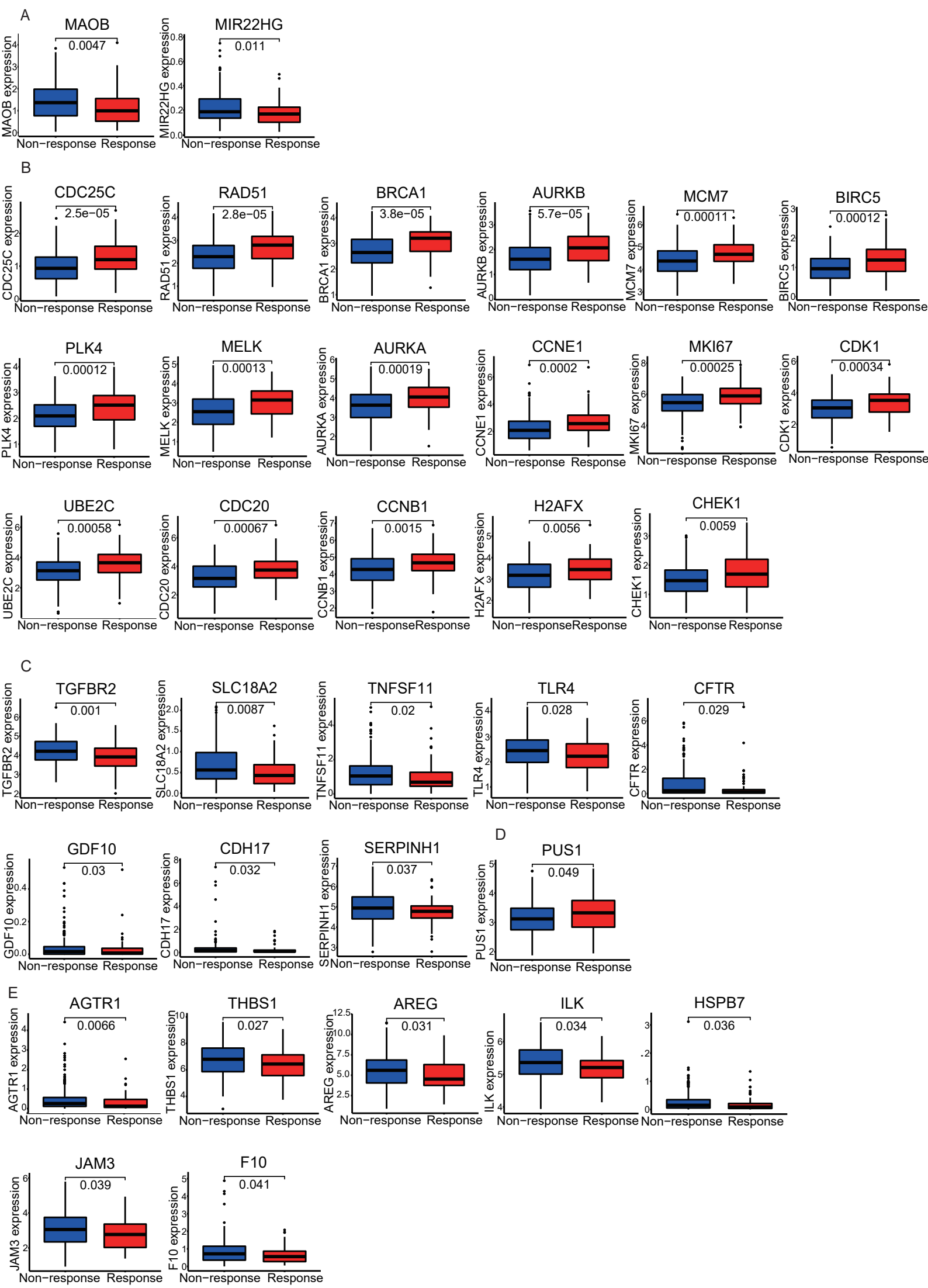

Supplement: Supplementary Materials — Table S1. The lung cancer-related pathways, therapy-related pathways, and other cancer-related KEGG pathways in LUAD. Table S2. The lung cancer-related pathways, therapy-related pathways, and other cancer-related KEGG pathways in LUSC. Figure S1. Immune checkpoint sensitivity exploration in the GSE126044 NSCLC cohort using prognostic curcumin-LUAD and curcumin-LUSC intersection genes. Figure S2. Potential genes related to immunotherapy response in the IMvigor210 cohort using prognostic curcumin-LUAD and curcumin-LUSC intersection genes. [file 5926609.f1.zip › Figure Supplementary.pdf]
