# Supplementary material for: The Pharmacological Mechanism of Curcumin against Drug Resistance in Non-Small Cell Lung Cancer: Findings of Network Pharmacology and Bioinformatics Analysis
Source: Evid Based Complement Alternat Med. 2022 Oct 14;2022:5926609. doi: 10.1155/2022/5926609 (PMC9586741; doi:10.1155/2022/5926609)
Supplement: Supplementary Materials — Table S1. The lung cancer-related pathways, therapy-related pathways, and other cancer-related KEGG pathways in LUAD. Table S2. The lung cancer-related pathways, therapy-related pathways, and other cancer-related KEGG pathways in LUSC. Figure S1. Immune checkpoint sensitivity exploration in the GSE126044 NSCLC cohort using prognostic curcumin-LUAD and curcumin-LUSC intersection genes. Figure S2. Potential genes related to immunotherapy response in the IMvigor210 cohort using prognostic curcumin-LUAD and curcumin-LUSC intersection genes. [file 5926609.f1.zip › Supplementary material20220924.doc]

**Supplementary material**

# The pharmacological mechanism of curcumin against drug resistance in non-small cell lung cancer: Findings of network pharmacology and bioinformatics analysis

#### Han Xie^1, 2, 3, 4, 5†^, Guirui Huang^6†^**, Jian**hua Zou^1, 2, 3, 4, 5^, Qianru Zhu^1, 2, 3, 4, 5^, Zimao Liang^1, 2, 3, 4, 5^, Xingxing Huang^1, 2, 3, 4, 5^, Xiangyang Zhai^1, 2, 3, 4, 5^, Ruonan Zhang^1, 2, 3, 4, 5^, Bi Chen^1, 2, 3, 4, 5^, Keshuai Li^1, 2^, Xinbing Sui^1, 2, 3, 4, 5^, Lili Yu^1*^, Hongsheng Cui^6*^, Qibiao Wu^1, 7,8,9*^

^1^ State Key Laboratory of Quality Research in Chinese Medicines, Faculty of Chinese Medicine, Macau University of Science and Technology, Wai Long, Macau;

^2^ School of Pharmacy and Department of Medical Oncology, The Affiliated Hospital of Hangzhou Normal University, School of Medicine, Hangzhou Normal University, Hangzhou, 311121, Zhejiang, China;

^3^ Key Laboratory of Elemene Class Anti-Cancer Chinese Medicines, Hangzhou, China;

^4^ Engineering Laboratory of Development and Application of Traditional Chinese Medicines, Hangzhou, China;

^5^ Collaborative Innovation Center of Traditional Chinese Medicines of Zhejiang Province, Hangzhou Normal University, Hangzhou 311121, Zhejiang, China;

^6^ Department of Respiratory, The Third Affiliated Hospital, Beijing University of Chinese Medicine, Beijing, China;

^7^ Guangdong-Hong Kong-Macao Joint Laboratory for Contaminants Exposure and Health, Guangdong University of Technology, Guangzhou 510006, China;

^8^ Zhuhai MUST Science and Technology Research Institute, Zhuhai 519000, Guangdong, China.

^9^ Zhuhai Hospital of Integrated Traditional Chinese and Western Medicine, Zhuhai 519000, Guangdong, China.

^†^ These authors made equal contributions to this work.

^*^ Corresponding authors: Lili Yu, Hongsheng Cui and Qibiao Wu

**Table S1.** The lung cancer-related pathways, therapy-related pathways, and other cancer-related KEGG pathways in LUAD.

|  | **Term ID** | **Term description** | **q value** |
| --- | --- | --- | --- |
| Lung cancer- related pathways | hsa04657 | IL-17 signaling pathway | 4.63E-05 |
|  | hsa04380 | Osteoclast differentiation | 0.000180976 |
|  | hsa04218 | Cellular senescence | 0.000498642 |
|  | hsa04668 | TNF signaling pathway | 0.000498642 |
|  | hsa05202 | Transcriptional misregulation in cancer | 0.000510507 |
|  | hsa04066 | HIF-1 signaling pathway | 0.0009826 |
|  | hsa04614 | Renin-angiotensin system | 0.0009826 |
|  | hsa04926 | Relaxin signaling pathway | 0.001359624 |
|  | hsa04540 | Gap junction | 0.005624078 |
|  | hsa04350 | TGF-beta signaling pathway | 0.008306151 |
|  | hsa04514 | Cell adhesion molecules | 0.043201721 |
| Therapy-related pathways | hsa04110 | Cell cycle | 0.000498642 |
|  | hsa04064 | NF-kappa B signaling pathway | 0.000637283 |
|  | hsa04010 | MAPK signaling pathway | 0.003939581 |
|  | hsa04151 | PI3K-Akt signaling pathway | 0.003939581 |
|  | hsa04115 | p53 signaling pathway | 0.005624078 |
|  | hsa01521 | EGFR tyrosine kinase inhibitor resistance | 0.008507401 |
|  | hsa04659 | Th17 cell differentiation | 0.01811746 |
|  | hsa02010 | ABC transporters | 0.01811746 |
|  | hsa04152 | AMPK signaling pathway | 0.031926146 |
|  | hsa01524 | Platinum drug resistance | 0.041107666 |
|  | hsa04060 | Cytokine-cytokine receptor interaction | 0.043201721 |
| Other cancer-related pathways | hsa05166 | Human T-cell leukemia virus 1 infection | 9.82E-05 |
|  | hsa05215 | Prostate cancer | 0.003939581 |
|  | hsa05219 | Bladder cancer | 0.013250988 |
|  | hsa05226 | Gastric cancer | 0.02312229 |
|  | hsa05221 | Acute myeloid leukemia | 0.031917336 |

**Table S2.** The lung cancer-related pathways, therapy-related pathways, and other cancer-related KEGG pathways in LUSC.

|  | **Term ID** | **Term description** | **q value** |
| --- | --- | --- | --- |
| Lung cancer- related pathways | hsa04668 | TNF signaling pathway | 1.44E-07 |
|  | hsa05202 | Transcriptional misregulation in cancer | 1.04E-06 |
|  | hsa04380 | Osteoclast differentiation | 1.12E-06 |
|  | hsa04510 | Focal adhesion | 1.63E-05 |
|  | hsa04657 | IL-17 signaling pathway | 0.000141303 |
|  | hsa04540 | Gap junction | 0.000239318 |
|  | hsa04066 | HIF-1 signaling pathway | 0.000542727 |
|  | hsa04218 | Cellular senescence | 0.001080362 |
|  | hsa04514 | Cell adhesion molecules | 0.016075019 |
|  | hsa04350 | TGF-beta signaling pathway | 0.018947503 |
| Therapy-related pathways | hsa04064 | NF-kappa B signaling pathway | 1.45E-08 |
|  | hsa04010 | MAPK signaling pathway | 1.12E-06 |
|  | hsa04151 | PI3K-Akt signaling pathway | 1.66E-06 |
|  | hsa04115 | p53 signaling pathway | 3.41E-05 |
|  | hsa04110 | Cell cycle | 3.41E-05 |
|  | hsa04640 | Hematopoietic cell lineage | 6.92E-05 |
|  | hsa04060 | Cytokine-cytokine receptor interaction | 9.68E-05 |
|  | hsa04620 | Toll-like receptor signaling pathway | 0.000373696 |
|  | hsa01524 | Platinum drug resistance | 0.000477967 |
|  | hsa01521 | EGFR tyrosine kinase inhibitor resistance | 0.000930113 |
|  | hsa04659 | Th17 cell differentiation | 0.008401393 |
|  | hsa02010 | ABC transporters | 0.008482266 |
| Other cancer-related pathways | hsa05166 | Human T-cell leukemia virus 1 infection | 3.18E-05 |
|  | hsa05221 | Acute myeloid leukemia | 0.000847046 |
|  | hsa05215 | Prostate cancer | 0.004024266 |
|  | hsa05219 | Bladder cancer | 0.005443934 |

**Figure S1.** Immune checkpoint sensitivity exploration in GSE126044 NSCLC cohort using prognostic curcumin-LUAD and curcumin-LUSC intersection genes. The results were exhibited below except for the genes showed in Figure 5A and 5B. These genes were divided into two parts based on their positive and negative associations with sensitivity and displayed in order of *p* value from lowest to highest. The x-coordinate represents response or non-response, and the y-coordinate shows the gene expression. **(A, B)** Genes that both expressed in curcumin-LUAD and curcumin-LUSC intersection gene sets. **(C, D)** Genes that expressed in curcumin-LUAD intersection gene set. **(E, F)** Genes that expressed in curcumin-LUSC intersection gene set.

**Figure S2.** Potential genes related to immunotherapy response in the IMvigor210 cohort using prognostic curcumin-LUAD and curcumin-LUSC intersection genes. The results were demonstrated below excluding the genes listed in Figure 5C and 5D. On the basis of their positive and negative relationships with sensitivity, these genes were separated into two groups and arranged from lowest to highest p value. The x-coordinate indicates response or non-response, and the y-coordinate is the gene expression. **(A)** Genes that both expressed in curcumin-LUAD and curcumin-LUSC intersection gene sets. **(B, C)** Genes that expressed in curcumin-LUAD intersection gene set. **(D, E)** Genes that expressed in curcumin-LUSC intersection gene set.
