# Supplementary material for: The Pharmacological Mechanism of Curcumin against Drug Resistance in Non-Small Cell Lung Cancer: Findings of Network Pharmacology and Bioinformatics Analysis
Source: Evid Based Complement Alternat Med. 2022 Oct 14;2022:5926609. doi: 10.1155/2022/5926609 (PMC9586741; doi:10.1155/2022/5926609)
Supplement: Supplementary Materials — Table S1. The lung cancer-related pathways, therapy-related pathways, and other cancer-related KEGG pathways in LUAD. Table S2. The lung cancer-related pathways, therapy-related pathways, and other cancer-related KEGG pathways in LUSC. Figure S1. Immune checkpoint sensitivity exploration in the GSE126044 NSCLC cohort using prognostic curcumin-LUAD and curcumin-LUSC intersection genes. Figure S2. Potential genes related to immunotherapy response in the IMvigor210 cohort using prognostic curcumin-LUAD and curcumin-LUSC intersection genes. [file 5926609.f1.zip › Tables20220924.doc]

**Table S1.** The lung cancer-related pathways, therapy-related pathways, and other cancer-related KEGG pathways in LUAD.

|  | **Term ID** | **Term description** | **q value** |
| --- | --- | --- | --- |
| Lung cancer- related pathways | hsa04657 | IL-17 signaling pathway | 4.63E-05 |
|  | hsa04380 | Osteoclast differentiation | 0.000180976 |
|  | hsa04218 | Cellular senescence | 0.000498642 |
|  | hsa04668 | TNF signaling pathway | 0.000498642 |
|  | hsa05202 | Transcriptional misregulation in cancer | 0.000510507 |
|  | hsa04066 | HIF-1 signaling pathway | 0.0009826 |
|  | hsa04614 | Renin-angiotensin system | 0.0009826 |
|  | hsa04926 | Relaxin signaling pathway | 0.001359624 |
|  | hsa04540 | Gap junction | 0.005624078 |
|  | hsa04350 | TGF-beta signaling pathway | 0.008306151 |
|  | hsa04514 | Cell adhesion molecules | 0.043201721 |
| Therapy-related pathways | hsa04110 | Cell cycle | 0.000498642 |
|  | hsa04064 | NF-kappa B signaling pathway | 0.000637283 |
|  | hsa04010 | MAPK signaling pathway | 0.003939581 |
|  | hsa04151 | PI3K-Akt signaling pathway | 0.003939581 |
|  | hsa04115 | p53 signaling pathway | 0.005624078 |
|  | hsa01521 | EGFR tyrosine kinase inhibitor resistance | 0.008507401 |
|  | hsa04659 | Th17 cell differentiation | 0.01811746 |
|  | hsa02010 | ABC transporters | 0.01811746 |
|  | hsa04152 | AMPK signaling pathway | 0.031926146 |
|  | hsa01524 | Platinum drug resistance | 0.041107666 |
|  | hsa04060 | Cytokine-cytokine receptor interaction | 0.043201721 |
| Other cancer-related pathways | hsa05166 | Human T-cell leukemia virus 1 infection | 9.82E-05 |
|  | hsa05215 | Prostate cancer | 0.003939581 |
|  | hsa05219 | Bladder cancer | 0.013250988 |
|  | hsa05226 | Gastric cancer | 0.02312229 |
|  | hsa05221 | Acute myeloid leukemia | 0.031917336 |

**Table S2.** The lung cancer-related pathways, therapy-related pathways, and other cancer-related KEGG pathways in LUSC.

|  | **Term ID** | **Term description** | **q value** |
| --- | --- | --- | --- |
| Lung cancer- related pathways | hsa04668 | TNF signaling pathway | 1.44E-07 |
|  | hsa05202 | Transcriptional misregulation in cancer | 1.04E-06 |
|  | hsa04380 | Osteoclast differentiation | 1.12E-06 |
|  | hsa04510 | Focal adhesion | 1.63E-05 |
|  | hsa04657 | IL-17 signaling pathway | 0.000141303 |
|  | hsa04540 | Gap junction | 0.000239318 |
|  | hsa04066 | HIF-1 signaling pathway | 0.000542727 |
|  | hsa04218 | Cellular senescence | 0.001080362 |
|  | hsa04514 | Cell adhesion molecules | 0.016075019 |
|  | hsa04350 | TGF-beta signaling pathway | 0.018947503 |
| Therapy-related pathways | hsa04064 | NF-kappa B signaling pathway | 1.45E-08 |
|  | hsa04010 | MAPK signaling pathway | 1.12E-06 |
|  | hsa04151 | PI3K-Akt signaling pathway | 1.66E-06 |
|  | hsa04115 | p53 signaling pathway | 3.41E-05 |
|  | hsa04110 | Cell cycle | 3.41E-05 |
|  | hsa04640 | Hematopoietic cell lineage | 6.92E-05 |
|  | hsa04060 | Cytokine-cytokine receptor interaction | 9.68E-05 |
|  | hsa04620 | Toll-like receptor signaling pathway | 0.000373696 |
|  | hsa01524 | Platinum drug resistance | 0.000477967 |
|  | hsa01521 | EGFR tyrosine kinase inhibitor resistance | 0.000930113 |
|  | hsa04659 | Th17 cell differentiation | 0.008401393 |
|  | hsa02010 | ABC transporters | 0.008482266 |
| Other cancer-related pathways | hsa05166 | Human T-cell leukemia virus 1 infection | 3.18E-05 |
|  | hsa05221 | Acute myeloid leukemia | 0.000847046 |
|  | hsa05215 | Prostate cancer | 0.004024266 |
|  | hsa05219 | Bladder cancer | 0.005443934 |
